# Evaluation of Sympathetic Function and Anxiety Levels in Sleep-Deprived Subjects Compared to the Normal Population: A Cross-Sectional Analytical Study

**DOI:** 10.7759/cureus.110894

**Published:** 2026-06-15

**Authors:** Sanjay S, Arunkumar B, Karthiga J, Aruna Raju

**Affiliations:** 1 Medical School, Jawaharlal Institute of Postgraduate Medical Education and Research, Karaikal, IND; 2 Physiology, Jawaharlal Institute of Postgraduate Medical Education and Research, Karaikal, IND; 3 Physiology, Vinayaka Mission's Medical College and Hospital, Karaikal, IND; 4 Physiology, All India Institute of Medical Sciences, Kalyani, Kalyani, IND

**Keywords:** anxiety, blood pressure, cardiovascular risk, sleep deprivation, sympathetic activity

## Abstract

Background

Sleep deprivation is associated with autonomic imbalance and psychological disturbances. This study aimed to evaluate sympathetic function and anxiety levels in sleep-deprived individuals compared with controls.

Methods

A cross-sectional analytical study was conducted among 110 participants aged 18-40 years, divided into sleep-deprived (n = 55) and well-rested (n = 55) groups based on Pittsburgh Sleep Quality Index scores. Sympathetic activity was assessed using cold pressor and isometric handgrip tests, and anxiety was measured using the Generalized Anxiety Disorder-7 (GAD-7) scale.

Results

Sleep-deprived participants demonstrated significantly higher basal heart rate (79.19 ± 4.23 vs. 71.20 ± 4.30 bpm), systolic blood pressure (125.11 ± 4.76 vs. 117.00 ± 3.93 mmHg), and diastolic blood pressure (79.50 ± 7.50 vs. 74.00 ± 7.00 mmHg; p < 0.001). Sympathetic reactivity was greater in the cold pressor test (10.92 ± 2.20 vs. 4.20 ± 2.34 mmHg; p < 0.001) and handgrip test (11.36 ± 2.53 vs. 3.94 ± 2.46 mmHg; p < 0.001) in the sleep-deprived group. Anxiety scores were also elevated (13.50 vs. 4.00; p < 0.001) in individuals with sleep deprivation compared with the well-rested group.

Conclusion

Sleep deprivation significantly increases sympathetic activity and anxiety, highlighting the importance of adequate sleep in preventing cardiovascular and psychological risks.

## Introduction

Sleep is a vital physiological phenomenon essential for maintaining homeostasis. It plays a crucial role in regulating various functions of the body, including cardiovascular health, cognitive processes, and emotional well-being. Adequate sleep of 7-8 hours/day helps maintain overall physiological balance and health [1].

Sleep deprivation refers to the condition of not getting enough sleep. Sleep disorders impose a significant health burden on the Indian population. It is estimated that about 93% of the Indian population experiences some form of sleep disorder or deprivation [2]. Lack of sufficient sleep can lead to complications such as cardiovascular problems, cognitive deficits, and mood disorders [3].

Few studies have established an association between sleep deprivation and sympathovagal imbalance [4]. It has been observed that when an individual is sleep-deprived, their sympathetic nervous system activity (SNS) is heightened, leading to elevated levels of catecholamines such as adrenaline and noradrenaline [5]. This fight-or-flight response is designed for short-term stress but becomes detrimental when chronically activated due to sleep deprivation. Consequently, individuals may experience increased heart rate and blood pressure, putting extra strain on the cardiovascular system and increasing the risk of heart disease [3]. Furthermore, prolonged sympathetic activation can disrupt glucose metabolism and decrease insulin sensitivity, contributing to metabolic disorders such as diabetes [6].

Sleep deprivation is also closely linked to changes in mental health and emotional well-being [7]. Anxiety is a common psychological consequence of inadequate sleep. The combined effects of increased sympathetic activity and heightened anxiety create a vicious cycle in which stress and anxiety resulting from sleep deprivation further disrupt sleep patterns [8, 9].

Understanding the intricate relationship between SNS function and anxiety levels in sleep-deprived individuals is essential for comprehensively addressing the health implications of insufficient sleep. Therefore, this study was designed to evaluate sympathetic reactivity and anxiety levels in sleep-deprived individuals compared with well-rested controls, and to assess their potential impact on cardiovascular and psychological health.

## Materials and methods

A convenience sampling technique was employed for recruitment. The sample size was calculated using OpenEpi software version 3. The sample size was computed using the mean difference in diastolic blood pressure (DBP) response to the cold pressor test reported by Carter JR et al. [3] (effect size d ≈ 1.03), a two-tailed alpha (α) = 0.05, and power (1-β) = 80%. This yielded a minimum of 48 participants per group; 55 were recruited per group to account for potential attrition.

Convenience sampling was used. To mitigate potential selection bias arising from the inclusion of two distinct occupational groups, MBBS students and security personnel, eligibility was determined solely by Pittsburgh Sleep Quality Index (PSQI) score [10], irrespective of occupation. No preferential sampling was applied to either subgroup, and occupational distribution across the study and control groups was verified and is reported in the Results section.

Participants were classified as well-rested based on a PSQI score of ≤5. However, six participants in the control group had a borderline score of 6. Sensitivity analyses excluding these six participants did not materially alter the group differences in any outcome variable, and the conclusions remain unchanged.

Study procedure

After obtaining written informed consent, the study parameters were recorded in both the study and control groups. All assessments were conducted between 8:00 AM and 11:00 AM to control for circadian variation in sympathetic tone. Prior to testing, participants were given standardized pre-test instructions: they were required to abstain from caffeine, nicotine, and strenuous physical exercise for a minimum of 12 hours before the test session; alcohol within 24 hours was covered by the exclusion criteria. All sympathetic function tests were performed in a quiet, temperature-controlled room maintained at 22-24°C to minimize environmental confounders.

Recording of anthropometric and basal cardiovascular parameters

Height and body weight were recorded using a stadiometer (least count 0.1 cm) and an analogue weighing scale (calibrated; least count 0.5 kg). BMI was calculated using Quetelet's index. Blood pressure was measured using an automated blood pressure (BP) monitor (AccuSure 1490-A) (AccuSure BP Monitor, Model 1490-A; accuracy ±3 mmHg or ±2%, calibrated prior to the study period). Heart rate was simultaneously recorded from the same device.

Recording of sympathetic function tests

Cold Pressor Test 

The baseline BP was recorded after 5 minutes of rest. The subject was instructed to immerse their hand in cold water (approximately 4°C; water temperature was monitored continuously using a calibrated digital thermometer to maintain 4°C ± 0.5°C throughout the procedure) for 1 minute. BP was recorded at the end of 1 min, 2 min, and 5 min of the procedure. Change in DBP from the baseline value was assessed. The maximum change in DBP (ΔDBP) was used as the primary summary outcome variable for this test [11].

Isometric Handgrip Test

The basal BP was recorded after 5 minutes of rest. The subject was then instructed to maintain a sustained handgrip at 30% of their maximum voluntary contraction for 2 minutes, measured using a Jamar Plus+ Digital Hand Dynamometer. BP was measured at the end of 2 minutes and 5 minutes. Change in DBP from the basal value was assessed. The maximum ΔDBP was used as the primary summary outcome variable for this test [11].

Assessment of anxiety score

The level of anxiety was assessed using the Generalized Anxiety Disorder-7 (GAD-7) questionnaire, a 7-item self-administered scale widely used to screen for and evaluate the severity of generalized anxiety disorder [12]. The GAD-7 was selected in preference to alternatives such as the Hamilton Anxiety Rating Scale (HAM-A) or the Anxiety Symptom Questionnaire (ASQ) for the following reasons: (1) it is a validated, freely available self-report tool with established reliability (Cronbach's α = 0.92) and excellent sensitivity (89%) and specificity (82%) for generalized anxiety disorder [12]; (2) unlike the clinician-administered HAM-A, the GAD-7 is suited for non-clinical, community-based research settings, reducing respondent burden; and (3) it has been validated for use in Indian populations, ensuring cultural applicability. Each item is scored on a 4-point Likert scale (0 = not at all, 1 = several days, 2 = more than half the days, 3 = nearly every day), yielding a total score ranging from 0 to 21. Scores are interpreted as follows: 0-4 = minimal anxiety; 5-9 = mild anxiety; 10-14 = moderate anxiety; 15-21 = severe anxiety.

The GAD-7 and PSQI questionnaires were self-administered in pen-and-paper format in the testing room prior to any physiological assessment. No investigator was present during completion to minimize social desirability bias. Participants were provided with written instructions and encouraged to seek clarification only for language comprehension issues. Completed forms were collected in sealed envelopes and de-identified before analysis.

Statistical tests used for data analysis

Normality of all continuous variables was assessed using the Shapiro-Wilk test and confirmed visually with Q-Q plots. The comparison of continuous data between the study group and control group was carried out using the independent samples t-test (parametric test, for normally distributed variables: heart rate, SBP, DBP, BMI, and age). Variables showing non-normal distribution (PSQI score and GAD-7 score, p < 0.05 on Shapiro-Wilk) were compared using the Mann-Whitney U test (non-parametric). Results are reported as mean ± SD for normally distributed data and as median (IQR) for non-normally distributed data. All statistical analyses were performed using IBM SPSS Statistics version 22 at the 5% level of significance; p-values < 0.05 were considered statistically significant.

## Results

The subjects in both groups were comparable in age and BMI. The occupational distribution was similar between groups: sleep-deprived group, MBBS students 38 (69.1%), security personnel 17 (30.9%); well-rested group, MBBS students 37 (67.3%), security personnel 18 (32.7%); χ² = 0.04, p = 0.84, indicating no significant difference. Sleep-deprived individuals had significantly higher PSQI scores compared with well-rested individuals, as shown in Table 1. Of the 55 control participants, 6 (10.9%) had a PSQI score of 6, which was borderline above the ≤5 threshold. Sensitivity analyses excluding these six participants did not materially alter the group differences in any outcome variable, and the conclusions remained unchanged.

**Table 1:** Demographic characteristics and sleep scores between the sleep-deprived and well-rested groups. Student’s t-test was used for age and BMI, and the Mann-Whitney U test was used for PSQI. *Statistically significant difference (p < 0.001). PSQI: Pittsburgh Sleep Quality Index.

Parameter	Sleep-deprived group (n = 55)	Well-rested group (n = 55)	Test value	p-value
Age (years)	20.33 ± 1.88	20.71 ± 1.86	-1.07	0.289
BMI (kg/m²)	22.18 ± 3.95	21.26 ± 3.12	1.36	0.178
PSQI score	8.00 ± 2.25	4.00 ± 2.00	9.135	<0.001*

At baseline, the sleep-deprived group showed a significantly higher basal HR compared with the well-rested subjects, as shown in Table 2. Basal SBP was also elevated in the sleep-deprived individuals, in addition to basal DBP, indicating elevated sympathetic activity even in the resting state.

**Table 2:** Basal cardiovascular parameters between the sleep-deprived and well-rested groups. HR: Heart rate; SBP: Systolic blood pressure; DBP: Diastolic blood pressure. Student’s t-test was used. *p < 0.001.

Parameter	Sleep-deprived group (n = 55)	Well-rested group (n = 55)	t-value	p-value	95% CI for difference	Cohen’s d
Basal HR (bpm)	79.19 ± 4.23	71.20 ± 4.30	9.82	< 0.001*	6.38-9.60	1.87 (large)
Basal SBP (mmHg)	125.11 ± 4.76	117.00 ± 3.93	9.74	< 0.001*	6.46-9.76	1.86 (large)
Basal DBP (mmHg)	79.50 ± 7.50	74.00 ± 7.00	3.98	< 0.001*	2.76-8.24	0.76 (medium)

The change in DBP in the cold pressor reactivity test was significantly greater in the sleep-deprived subjects compared with the well-rested group. Also, the magnitude of change in DBP during the handgrip test was significantly higher in the sleep-deprived group. Anxiety symptoms assessed using the GAD-7 scale were elevated in the sleep-deprived group compared with the well-rested group, as shown in Table 3.

**Table 3:** Sympathetic function test results and anxiety scores between the sleep-deprived and well-rested groups. CP: Cold pressor test; HG: Handgrip test; GAD-7: Generalized Anxiety Disorder-7; ΔDBP: Change in diastolic blood pressure. ΔDBP values were analyzed using the independent samples t-test, and GAD-7 scores were analyzed using the Mann-Whitney U test.

Parameter	Sleep-deprived group (n = 55)	Well-rested group (n = 55)	Test statistic	p-value	95% CI / effect size	Interpretation
ΔDBP cold pressor test (mmHg)	10.92 ± 2.20	4.20 ± 2.34	15.52	< 0.001*	95% CI: 5.86-7.58; d = 2.96	Very large
ΔDBP handgrip test (mmHg)	11.36 ± 2.53	3.94 ± 2.46	15.59	< 0.001*	95% CI: 6.48-8.36; d = 2.97	Very large
GAD-7 score, median (IQR)	13.50 (4.50)	4.00 (2.00)	Z = -8.43	<0.001*	r = 0.81	Large

## Discussion

This study aimed to evaluate the relationship between sleep deprivation, SNS activity, and anxiety levels in sleep-deprived individuals compared with well-rested individuals. The findings from this study show a significant increase in sympathetic function and anxiety levels in sleep-deprived subjects compared with individuals having adequate sleep.

Among the many factors that promote sympathovagal balance, sleep plays a crucial role. It is well established that the deep stages of sleep are associated with increased parasympathetic function, which helps in restoring energy and reducing stress [5]. Reduced sleep thereby disrupts this autonomic balance, which results in sympathetic overactivity. Elevated sympathetic activity has a profound influence on various cardiovascular parameters and has been implicated in the pathogenesis of various cardiovascular disorders [13]. Increases in heart rate, blood pressure, and altered sympathetic function test results in sleep-deprived subjects suggest that sleep deprivation can cause a state of autonomic imbalance that may lead to adverse health outcomes over time [5]. It is well established that chronic sympathetic activation may predispose individuals to various health issues, including metabolic and cardiovascular diseases [14]. This indicates the importance of maintaining sleep hygiene to improve the overall health of individuals.

The observed mean SBP of 125.11 ± 4.76 mmHg in the sleep-deprived group falls within the “elevated” category as per the 2017 ACC/AHA guidelines (120-129 mmHg), but does not meet the threshold for Stage 1 hypertension (≥130 mmHg). It is therefore important to distinguish between the acute physiological sympathoexcitatory response to sleep deprivation demonstrated in this study and the development of chronic, sustained hypertension. Our findings reflect short-term haemodynamic changes in a young, normotensive cohort and should not be interpreted as evidence of established cardiovascular disease. Rather, they highlight a mechanistic pathway: if sustained chronically, repeated sympathetic activation and elevated catecholamine levels may cumulatively contribute to endothelial dysfunction, arterial stiffness, and ultimately hypertension, as supported by longitudinal epidemiological evidence.

Additionally, anxiety levels in the sleep-deprived group were found to be elevated. This suggests that reduced sleep increases susceptibility to anxiety disorders, which supports the findings from previous studies [15]. Though many complex mechanisms are involved in the pathogenesis of anxiety disorders, one of the important pathways involved is the hypothalamic-pituitary-adrenal (HPA) axis [16]. Increased activation of the HPA axis in response to sleep deprivation leads to increased release of cortisol, which is the primary stress hormone. Elevated cortisol levels alter the neuronal circuits in the amygdala and prefrontal cortex, the key areas involved in emotion, thereby causing anxiety symptoms [17]. In addition, sleep deprivation alters the neurochemical balance, especially those involving serotonin, norepinephrine, and dopamine, which are involved in mood regulation [18].

Increased sympathetic activity contributes to anxiety, which further increases sympathetic activation, thus creating a vicious cycle [19]. Overall, this study indicates that sleep deprivation leads to heightened sympathetic activity, which has an adverse effect on cardiovascular and emotional health, highlighting the importance of adequate sleep as a vital factor in a healthy lifestyle.

Limitations

This study has several limitations that should be considered when interpreting its findings. The cross-sectional design prevents causal inference; the direction of observed associations cannot be definitively established. Longitudinal studies with repeated measures are required to confirm whether sleep deprivation causally precedes autonomic dysfunction or whether pre-existing sympathetic hyperreactivity predisposes individuals to poorer sleep. Sleep quality was assessed using the PSQI, a validated and widely used self-report questionnaire; however, subjective reporting carries an inherent risk of recall bias. The absence of objective sleep verification using actigraphy or polysomnography limits the precision of sleep categorization. Future studies should incorporate such measures to objectively quantify sleep deprivation. Convenience sampling limits the generalizability of findings beyond the study population.

Future directions

Future research should involve: (1) prospective longitudinal or interventional studies examining the effect of sleep hygiene interventions, such as cognitive behavioural therapy for insomnia, structured exercise programmes, or mindfulness-based stress reduction, specifically on sympathetic reactivity measured by standardized autonomic function tests; (2) incorporation of biochemical markers, particularly salivary or urinary cortisol and plasma catecholamines, to provide a more holistic assessment of HPA axis activation and sympathoadrenal response; and (3) heart rate variability (HRV) analysis as a continuous, objective, non-invasive index of sympathovagal balance to complement the challenge-based tests employed in this study.

## Conclusions

Adequate sleep is essential for physical and mental well-being. The observed findings from this study show that SNS activity and anxiety levels increase significantly in sleep-deprived individuals, emphasizing the need for adequate sleep in autonomic regulation and mental well-being. For individuals experiencing chronic sleep deprivation, lifestyle modifications, including regular aerobic exercise and mindfulness-based practices, may help restore sympathovagal balance and attenuate anxiety symptoms. Routine assessment of sleep habits during clinical encounters is recommended, as early identification and management of poor sleep hygiene may reduce long-term cardiovascular and psychological risks.
